# BST-2 controls T cell proliferation and exhaustion by shaping the early distribution of a persistent viral infection

**DOI:** 10.1371/journal.ppat.1007172

**Published:** 2018-07-20

**Authors:** Shuzo Urata, Elizabeth Kenyon, Debasis Nayak, Beatrice Cubitt, Yohei Kurosaki, Jiro Yasuda, Juan C. de la Torre, Dorian B. McGavern

**Affiliations:** 1 National Research Center for the Control and Prevention of Infectious Diseases (CCPID), Nagasaki University, Nagasaki, Japan; 2 Department of Emerging Infectious Diseases, Institute of Tropical Medicine, Nagasaki University, Nagasaki, Japan; 3 Department of Immunology and Microbial Science IMM-6, The Scripps Research Institute, La Jolla, California, United States of America; 4 Viral Immunology & Intravital Imaging Section, National Institute of Neurological Disorders and Stroke, National Institutes of Health, Bethesda, Maryland, United States of America; 5 Center for Bioscience and Biomedical Engineering, Indian Institute of Technology Indore, India; University of Pennsylvania, UNITED STATES

## Abstract

The interferon inducible protein, BST-2 (or, tetherin), plays an important role in the innate antiviral defense system by inhibiting the release of many enveloped viruses. Consequently, viruses have evolved strategies to counteract the anti-viral activity of this protein. While the mechanisms by which BST-2 prevents viral dissemination have been defined, less is known about how this protein shapes the early viral distribution and immunological defense against pathogens during the establishment of persistence. Using the lymphocytic choriomeningitis virus (LCMV) model of infection, we sought insights into how the *in vitro* antiviral activity of this protein compared to the immunological defense mounted *in vivo*. We observed that BST-2 modestly reduced production of virion particles from cultured cells, which was associated with the ability of BST-2 to interfere with the virus budding process mediated by the LCMV Z protein. Moreover, LCMV does not encode a BST-2 antagonist, and viral propagation was not significantly restricted in cells that constitutively expressed BST-2. In contrast to this very modest effect in cultured cells, BST-2 played a crucial role in controlling LCMV *in vivo*. In BST-2 deficient mice, a persistent strain of LCMV was no longer confined to the splenic marginal zone at early times post-infection, which resulted in an altered distribution of LCMV-specific T cells, reduced T cell proliferation / function, delayed viral control in the serum, and persistence in the brain. These data demonstrate that BST-2 is important in shaping the anatomical distribution and adaptive immune response against a persistent viral infection *in vivo*.

## Author summary

BST-2 (or, tetherin) is an antiviral protein that inhibits the release and spread of many enveloped viruses and is upregulated as part of the innate immune defense against infections. By studying the lymphocytic choriomeningitis virus (LCMV) model of infection, we sought insights into how BST-2 shapes the early viral distribution and immunological defense against a virus during the establishment of persistence. We also studied how the antiviral activity of BST-2 in cell culture compared to the immunological defense mounted by this protein in LCMV-infected mice (*in vivo*). We observed that BST-2 only modestly affected the production of LCMV in cultured cells by interfering with the viral budding process, but played a crucial role in controlling LCMV *in vivo*. In the absence of BST-2, a persistent strain of LCMV was ineffectively contained in the spleen at early time points post-infection, resulting in impaired antiviral T cell responses and viral control systemically. These data demonstrate that BST-2 is important in shaping the anatomical distribution and adaptive immune response against a persistent viral infection *in vivo*.

## Introduction

Arenaviruses are enveloped viruses with a bi-segmented, negative strand RNA genome and a life cycle restricted to the cell cytoplasm [1]. Each genome segment uses an ambisense coding strategy to direct the expression of two proteins in opposite orientation and separated by a non-coding intergenic region. The large segment encodes for the RNA-dependent-RNA-polymerase (L) and the matrix protein (Z) that mediates viral assembly and budding [2–4]. The small segment (S; 3.5 kb) encodes the glycoprotein (GP) precursor, GPC, and the viral nucleoprotein (NP). GPC is co-translationally cleaved by signal peptidase to produce a stable 58 amino acid Stable Signal Peptide (SSP) and GPC that is post-translationally processed by the cellular Site 1 Protease to yield the two mature virion glycoproteins (GP1 and GP2) that together with SSP form the GP complex involved in receptor binding and virus cell entry. GP1 mediates virion attachment to the cell surface followed by cell entry via receptor-mediated endocytosis, whereas GP2 is responsible for the pH-dependent fusion event in the acidic environment of the endosome to complete the virus cell entry process and release of virus ribonucleoprotein into the cell cytoplasm to initiate transcription and replication of the viral genome [1]. The L polymerase and NP are the minimal trans-acting factors required for virus RNA replication and gene transcription [5], whereas production of infectious particles also requires GP and Z [2].

Several arenaviruses cause hemorrhagic fevers in humans [1]. Thus, Lassa (LASV) and Junin (JUNV) viruses, the causative agents of Lassa fever and Argentine hemorrhagic fever [6], respectively, pose important public health concerns within their endemic regions of West Africa (LASV) and Argentina (JUNV). Notably, increased travel has resulted in importation of Lassa fever cases into non-endemic regions [7]. Moreover, evidence indicates that the globally distributed prototypic arenavirus lymphocytic choriomeningitis virus (LCMV) is a neglected human pathogen of clinical significance, especially in congenital viral infections [8]. Besides their impact on public health, arenaviruses also pose a credible bioterrorism threat, and six of them, including LASV and JUNV, have been classified as Category A agents. Concerns about arenavirus infections of humans are exacerbated by a limited availability of arenavirus countermeasures. The live attenuated Candid#1 strain of JUNV was shown to be an effective vaccine against Argentine hemorrhagic fever [9], but Candid#1 is licensed exclusively in Argentina and does not protect against Lassa fever. Likewise, current anti-arenaviral drug therapy is restricted to an off label use of the nucleoside analogue ribavirin, which is only partially effective and associated with significant side effects [10]. Therefore, there is an unmet need to identify novel compounds that could be developed into FDA-approved antiviral drugs to combat human pathogenic arenaviruses—a task that would be facilitated by a better understanding of virus-host interactions that regulate different steps of the arenavirus life cycle.

BST-2 (a.k.a. tetherin, CD317, or HM1.24) is a type I interferon (IFN-I)-inducible cellular protein that was initially identified as an inhibitor of HIV-1 release and was subsequently shown to inhibit cell release of a wide range of enveloped viruses including LASV [11, 12]. Several viral gene products, including HIV-1 Vpu and Ebola virus GP [13–16], were demonstrated to counteract the antiviral activity of BST-2. Here, we sought insights into whether BST-2 interferes with the release and propagation of the prototypic arenavirus, LCMV, both in cultured cells and during the establishment of a persistent viral infection in mice. Our results demonstrate how a relatively modest antiviral effect *in vitro* can translate into a large impact on antiviral immunity *in vivo*.

## Materials and methods

### Ethics statement

All mice in this study were handled in accordance with the guidelines set forth by the NIH Animal Care and Use Committee.

### Mice

C57BL/6J (B6) mice were purchased from The Jackson Laboratory. BST-2 KO [17] (provided by Dr. Marco Colonna, Washington University), P14 [18], Thy1.1^+^ P14, mOrange^+^ P14 [19], SMARTA [20], and CD45.1^+^ SMARTA, (all on a pure B6 background) were bred and maintained under specific pathogen–free conditions at the National Institutes of Health (NIH).

### Plasmids, antibodies and cells

Plasmids pC-LCMV-Z-FLAG, pTeth-FL, pGFP, pCEboZVP40 [21–25], as well as p-T7, pMG-CAT, pCAGGS-NP, and pCAGGS-L [5, 26–29] have been described. Plasmid psiCHECK2 was purchased from Promega (Madison, WI). The mouse monoclonal antibody (MAb) to the FLAG epitope was purchased from SIGMA (M2, St. Louis, MO). The mouse MAb to LCMV NP has been described [30]. Anti-human BST-2 polyclonal antibody was provided from NIH AIDS Reagent Program (Catalog number: 11721, provided by Drs. Klaus Strebel and Amy Andrew) [31]. Anti-Ebola virus VP40 polyclonal antibody was described previously [23]. HeLa-pLKO (control HeLa cell line), HeLa-TKD (BST-2 stably knocked down HeLa cell line), 293T, BHK-21, VeroE6, Huh7.5.1 and Huh7.5.1/BST2 (Huh7.5.1 constitutively expressing high levels of BST-2) cells were grown in Dulbecco’s modified Eagle’s medium (DMEM; Invitrogen, Carlsbad, CA) containing 10% fetal bovine serum (FBS), 1% penicillin and streptomycin. To generate Huh7.5.1/BST2 cells, we cloned the human BST-2 gene into a lentiviral vector that expressed also, via an IRES sequence, the gene of resistance to puromycin. We produced VSV-G pseudotyped lentiviral particles expressing BST-2 by co-transfecting HEK293T cells with HIV-1 packaging plasmids and the lentiviral vector expressing BST-2. At 48 hours post-transfection, we collected and concentrated lentiviral particles present in tissue culture supernatant. We used dilutions of the prep of BST-2 expressing lentiviral particles to infect Huh 7.5.1 cells to determine the dilution that resulted in 50% of cells expressing BST-2 at 72 hours post-transduction. This dilution was used to transduce Huh 7.5.1 cells followed by selection in the presence of puromycin (8 μg/ml). BST-2 expression in puromycin selected cells was determined by Western blot using an antibody to BST-2. Anti-actin mouse monoclonal antibody (AC-40, Sigma) was used to detect actin. Lentivirus transduced cells expressing BST-2 were further selected by FACS to select a population of cells expressing high levels of BST-2, called Huh-7.5.1/BST-2. HeLa-pLKO, HeLa-TKD and Huh7.5.1/BST2 cells were maintained in the presence of puromycin (8 μg/ml).

### VLP assay

2.5x10^5^ 293T cells were transfected with 0.2 μg of pC-LCMV-Z-FLAG using LT-1 (3 μl LT-1/μg DNA, Mirus). At 48 hrs post-transfection, VLP-containing tissue culture supernatants (TCS) and cells were collected. After clarification from cell debris (1,500 x g; 5 min), VLPs were collected by ultracentrifugation (100,000 x g; 30 min at 4°*C*) through a 20% sucrose cushion. Cells and VLPs were re-suspended in lysis buffer (1% NP-40, 50 mM Tris-HCl [pH 8.0], 62.5 mM EDTA, 0.4% sodium deoxycholate) and analyzed by Western blot. For Ebola virus (EBOV) VP40-mediated VLPs, 293T cells were transfected with 0.1 μg of pEboZVP40 for 5 h, followed by infection with rLCMV/Z-FLAG (moi = 5), or mock-infected. At 16 hrs p.i., VLP-containing TCS and cells were collected. After clarification from cell debris (1,500 x g; 5 min), VLPs were collected by ultracentrifugation (345,000 x g for Ebola VLPs; 100,000 x g for LCM VLPs; 30 min at 4°*C*) through a 20% sucrose cushion. Cell lysates and VLPs were prepared and analyzed by WB. Bands corresponding to Z and VP40 were quantified using Multi Gauge software (Ver2.0, Fuji Film).

### Immunoblotting

Cell lysates or VLP samples prepared as described above, were resolved by SDS-PAGE followed by Western blot using the indicated antibodies. Flag-tagged Z and BST2 proteins were detected with an anti-Flag mouse monoclonal antibody, and antigen-antibody complexes revealed using an HRP-conjugated anti-mouse IgG antibody. Endogenous BST-2 was detected using a rabbit polyclonal anti-BST2 serum, followed by an HRP-conjugated anti-rabbit IgG antibody. Ebola virus VP40 was detected using a rabbit polyclonal antibody to VP40, followed by an HRP-conjugated anti-rabbit IgG antibody. Actin, used as loading control, was detected using a mouse monoclonal antibody to actin, followed by an HRP-conjugated anti-mouse IgG antibody. ECL prime (GE healthcare) and LAS3000 (GE healthcare) were used to detect labeled proteins.

### Northern blot

Total cellular RNA was isolated using TRI Reagent (Molecular Research Center, Inc, Cincinnati, OH) per the manufacturer’s instructions, and isolated RNA was analyzed by Northern blot hybridization. Briefly, RNA samples were fractionated by 2.2 M formaldehyde-agarose (1.2%) gel electrophoresis followed by transfer (4 hrs) in 20 X SSC of the RNA to a Magnagraph (0.22 μm) membrane using the rapid downward transfer system (TurboBlotter). Membrane bound RNA was crosslinked by exposure to UV and the membrane was hybridized to a ^32^P-labeled strand specific probe to the MG-derived CAT mRNA.

### Viruses, virus infection and virus titration

The Armstrong (Arm) and Clone 13 (Cl-13) strains of LCMV [32, 33] as well as tri-segment recombinant LCMV (3rLCMV/GFP) [21, 34, 35] and rLCMV/Z-FLAG [35] have been described. Mice were infected intravenously with 2x10^6^ PFU of LCMV Arm or Cl-13. LCMV titers were determined using an immunofocus assay [36] or by plaque assay using Vero cells. For the immunofocus assay, 10-fold serial virus dilutions were used to infect Vero cell monolayers in a 96-well plate, and at 20 hrs p.i., cells were fixed with 4% paraformaldehyde (PFA) in PBS. After cell permeabilization by treatment with 0.3% Triton X-100 in PBS containing 3% BSA, cells expressing viral antigen were stained by using a mouse MAb to LCMV NP and an Alexa Fluor 568-labeled anti-mouse second antibody (Molecular Probes, Eugene, OR). For the plaque assay, 10-fold serial virus dilutions were used to infect Vero cells in M-24 well plates. After 90 min adsorption, the virus inoculum was removed, and cell monolayers were washed once and overlaid with DMEM containing 0.5% agarose, 1% Glutamine, 1% penicillin and streptomycin, and 0.7% FBS. After six days incubation, cells were fixed with 4% PFA and plaques were visualized by crystal violet staining. Titers of vesicular stomatitis Indiana virus (VSV) were determined by plaque assay using BHK-21 cells or Vero cells.

### Mini-genome (MG) assay

293T cells were seeded (4.5 x 10^5^/well) on M-12 well plates and the following day transfected with p-T7, pMG-CAT, pCAGGS-NP, pCAGGS-L, and either pTeth-FL or pGFP under the conditions described [5, 26–29]. At 24 hrs post-transfection cell lysates were prepared to determine levels of CAT protein by ELISA using a CAT ELISA kit (Roche, Basel, Switzerland) and using an ELISA reader (SPECTRA max plus 384, Molecular Devices, Sunnyvale, CA) to determine the absorbance (405 nm for samples, 490 nm for the reference).

### Pol-II based transcription assay

293T cells were seeded (5x10^4^/well) on 96 well plate and transfected with psiCHECK2 (100 ng/well) and indicated amount of pTeth-FL using LT-1. Total plasmid volume was adjusted with pcDNFL (empty) vector. At 48 hrs post-transfection cell lysates were prepared for Dual-Glo Reporter Assay (Promega) to detect both Firefly luciferase (Fluc) and Renilla Luciferase (Rluc), respectively, according to the manufacturer’s recommendations. Values of luciferase activity were measured using a luminometer (Centro LB 960, Berthold technologies, Bad Wildbad, Germany). Luciferase activity values were normalized assigning 1.0 to those obtained with lysates from vehicle (DMSO)-treated cells.

### Cell viability assay

Cell viabilities of increasing amounts of exogenous BST-2 expression in 293T cells were assessed using the CellTiter-Glo Luminescent Cell Viability Assay (Promega). The assay was performed according to the manufacturer’s recommendations and readings were obtained using a luminometer (Centro LB 960). Cell viability of control plasmid transfected cells was normalized and set at 1.0.

### Immunofluorescence assay (IFA)

HeLa cells infected with rLCMV/Z-FLAG were fixed using 4% PFA at 24 or 48 hrs p.i. and permeabilized by treatment with 0.3% Triton X-100 in PBS containing 3% BSA. Cells were stained using a mouse MAb to FLAG (M2) as the 1^st^ antibody for 2 hrs at room temperature (RT), followed by Alexa Fluor 568-labeled anti-mouse second antibody (Molecular Probes). To detect endogenous intracellular BST-2, cells were reacted with a rabbit polyclonal serum to BST-2 [31] for 2 hrs at RT, followed by an Alexa 488-conjugated anti-rabbit antibody for 2 hrs at RT. Cell nuclei were identified by DAPI staining. Samples were examined by laser confocal microscopy (LSM780 ELYRA system; Carl Zeiss, Oberkochen, Germany).

### Mononuclear cell isolations

Anesthetized mice received an intracardiac perfusion with PBS or saline to remove all contaminating blood lymphocytes. Single cell suspensions from spleen were prepared by mechanical disruption through a 100-μm strainer followed by red blood cell lysis with ammonium chloride buffer (0.017 M Tris-HCl and 0.14 M NH_4_Cl, pH 7.2).

### Adoptive transfers

For experiments involving transfer of Thy1.1+ P14, mOrange+ P14, and CD45.1+ SMARTA T cells, the respective populations were purified from the spleens of naive transgenic mice using negative selection kits (STEMCELL Technologies). For co-transfer experiments, naive recipient mice were seeded i.v. with 2,000 Thy1.1^+^ P14 and 2,000 CD45.1^+^ SMARTA T cells. For experiments to determine the anatomical distribution of antiviral CD8+ T cells, mice were seeded i.v. with 10,000 mOrange+ P14. Mice for both experimental lines were infected one day later with LCMV Cl-13.

### Flow cytometry

To detect cell surface expression of BST-2 *in vitro*, cells were washed with PBS once and treated with Accutase (AT104; Innovative Cell Technologies, Inc., San Diego, CA). Cells were collected in PBS containing 1% FBS and separated into two tubes for PE-conjugated mouse monoclonal control antibody (MOPC-21; BioLegend, San Diego, CA) or PE-conjugated mouse monoclonal anti-BST-2 antibody (RS38E; BioLegend) staining. After antibody staining, cells were fixed with 2% PFA for 15 min at RT and analyzed by FACS (Caliber, BD, San Jose, CA). For flow cytometry experiments involving splenocytes, cells were incubated for 20 min on ice with cocktails of mAbs in PBS containing 2% FBS. Before staining, all cell preparations were incubated with 3.3 μg/ml rat anti–mouse CD16/32 (Fc receptor block; BD) and 1:50 whole mouse IgG (Jackson ImmunoResearch Laboratories, Inc.) for 10 min on ice to reduce unspecific antibody binding. Dead cells were excluded from the analysis by using the LIVE/ DEAD fixable Blue Cell Staining kit (ThermoFisher Scientific). The following antibodies were obtained from BioLegend (BL), BD, or eBioscience (eB): B220 PE (RA3-6B2; BD), BST-2 Pacific Blue (927; BL), CD11b BV605 (M1/70; BL), CD11c PE/Cy7 (N418; BL), CD19 APC-Cy7 (6D5; BL), CD4 APC (RM4-5; BL), CD4 BV605 (RM4-5; BL), CD45.1 PECy7 (A20; BL), CD45.2 AF700 (104; BL), CD45.2 FITC (104; BD), CD45.2 BV421 (104; BL), CD8 BV510 (53–6.7; BL), CD8 FITC (53–6.7; BL), Thy1.2 AF700 (30-H12; BL), Thy1.1 PE (OX-7; BD), and Thy1.1 PECy7 (HIS51; eB). To detect intracellular cytokine production, single cell suspensions were surface stained as described above, treated with cytofix/cytoperm (BD), and then stained intracellularly with anti-IFN-γ PE (XMG1.2; BD), TNF-α FITC (MP6-XT22; BD), and IL-2 APC (JES6-5H4; BD). Samples were acquired using an LSRII flow cytometer (BD), and data were analyzed using FlowJo software version 10.0.7 (Tree Star).

### *In vitro* cytokine production

Two million splenocytes were plated in 96-well round bottom plates in RPMI complete media (RPMI; 10% FBS, 1% penicillin/streptomycin, 1% L-glutamine, 1% HEPES, 1% nonessential amino acids, 1% sodium pyruvate, 50 μM 2-mercaptoethanol, 1 μg/ml of Brefeldin A) with 2 μg/ml GP33-41 peptide *(*KAVYNFATC; Anaspec) [37] or 4 μg/ml GP61-80 peptide (GLNGPDIYKGVYQFKSVEFD; Anaspec) [38] at 37°C for five hours.

### *In vivo* cell proliferation

T cell proliferation was measured by carboxyfluorescein diacetate N-succinimidyl ester (CFSE; Molecular Probes) dilution. Naïve Thy1.1+ P14 cells were incubated for 10 min at 37°C in PBS containing 0.1% BSA and 5 μM CFSE. Following a wash, 5x10^5^ labeled T cells were injected i.v. into naïve mice, which were then infected with LCMV.

### Immunohistochemistry

For immunohistochemical experiments, mice were perfused with saline or 4% paraformaldehyde (PFA) in PBS. The latter was used for experiments involving fluorescent protein expressing transgenic P14 cells. Spleens extracted from PFA-perfused mice were incubated overnight in 4% PFA in PBS and then for an additional 24 hr in a 30% sucrose solution. Tissues were then frozen on dry ice in Tissue-tek optimal cutting temperature medium (Thermo Fisher Scientific). 6-μm cryosections were fixed for 10 min with 4% PFA, washed three times with PBS, blocked with avidin-biotin blocking kit (Vector Laboratories) per the manufacturer’s instructions, and stained with primary antibodies overnight at 4°C in PBS containing 2% FBS. The following were used as primary antibodies: rat anti-LCMV (1:1,000; clone VL-4; Bio X Cell), polyclonal anti-laminin (1:500; Abcam), biotinylated anti-BST-2 (1:500; clone 927; Biolegend), and anti-CD169-FITC (1:400; clone 3D6.112; Serotec). After washing three times with PBS, tissue sections were stained with species-specific fluorescently conjugated secondary antibodies (1:400; Jackson ImmunoResearch Laboratories, Inc.) or streptavidin rhodamine red X (1:400; Jackson ImmunoResearch Laboratories, Inc.) for 1 hr at RT, washed, and costained with 10 ng/ml DAPI (Sigma-Aldrich) to label cell nuclei. All slides were mounted with Vectashield (Vector Laboratories), and fluorescent images were acquired using a FV1200 confocal microscope (Olympus) equipped with an automated *xyz* stage, six laser lines (405, 458, 488, 515, 559, and 635 nm), and 4, 10, 20, and 40x objectives. In Fig 4D, the percentage of LCMV+ pixels in the splenic white versus red pulp were calculated by segmenting out the signal corresponding to LCMV staining. The segmented images were then used to calculate the total number of white vs. red pulp LCMV+ pixels. The LCMV+ pixel counts were divided by the white vs. red pulp pixel areas and multiplied by a hundred to generate percentages of tissue occupied by LCMV. In Fig 6B, the percentage of P14 cells in the white vs. red pulp was calculated by identifying fluorescently labeled cells in splenic images and then calculating the proportion that localized to these distinct anatomical regions.

## Results

### Effects of BST-2 on LCMV multiplication in cultured cells

**Fig 1:**
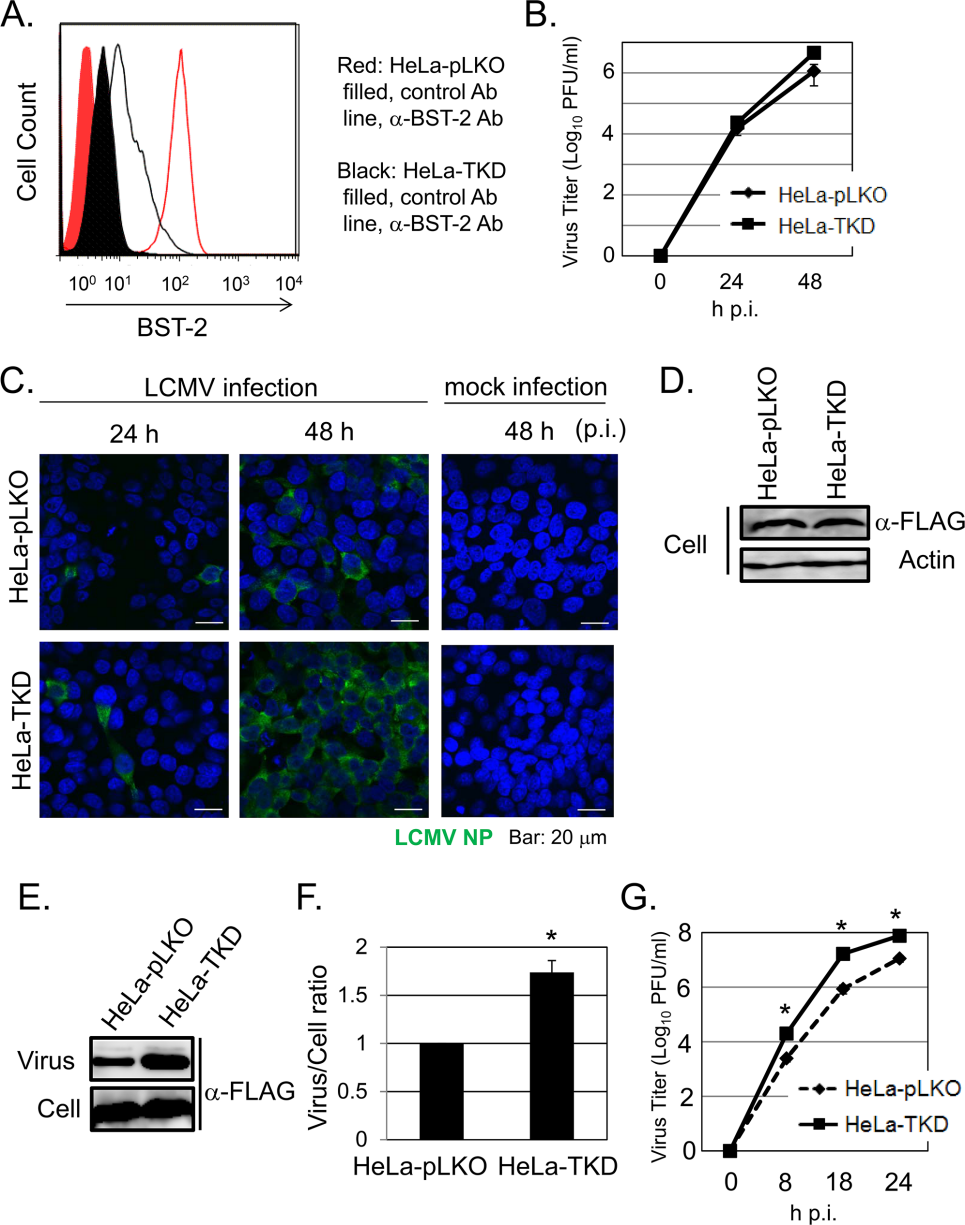
Endogenous BST-2 expression levels have a very limited impact on LCMV propagation *in vitro*. **A.** Cell surface expression of BST-2 in HeLa-TKD and HeLa-pLKO cells. Cells were fixed and analyzed by FACS using either a control antibody (Ab) or anti-BST-2 conjugated to PE. **B.** Growth kinetics of LCMV in HeLa-pLKO and HeLa–TKD cells. Cells were infected with LCMV (moi = 0.01). At 24 and 48 hrs p.i, virus titers in TCS were determined by plaque assay (n = 3; 2 independent experiments). **C.** Propagation of LCMV in HeLa-pLKO and HeLa-TKD cells. Cells were infected with LCMV (moi = 0.1). At 24 and 48 h p.i., cells were fixed with 4% PFA and after permeabilization stained with a MAb to LCMV NP, followed with a second antibody conjugated to FITC. Nuclei were stained with DAPI. Mock infected cells were used as control. **D.**Similar Z protein expression levels in HeLa-TKD and HeLa-pLKO cells at 16 h p.i. Cells were infected (moi = 0.1) with rLCMV/Z-FLAG and at 16 h p.i. expression levels of Z protein determined by WB using a MAb to FLAG. Levels of actin were used as loading control. **E-F.** Production of virion particles by LCMV-infected HeLa-TKD and HeLa-pLKO cells. Cells were infected with rLCMV/Z-FLAG (moi = 0.1) and at 24 hrs p.i. virion particles present in TCS were recovered by ultracentrifugation, and cell lysates prepared. Levels of Z protein present in virion particle preps and cell lysates were determined by WB using an Ab to FLAG (**E**), and signals quantified with LAS3000 (Fuji Film) (**F**). The ratio of virus/cell Z protein levels in HeLa-pLKO was set to 1.0 (n = 3; 3 independent experiments). **G.** Growth kinetics of VSV in HeLa-pLKO and HeLa–TKD cells. Cells were infected with VSV (moi = 0.01). At 8, 18 and 24 h p.i, virus titers in TCS were determined by plaque assay (n = 3; 2 independent experiments). Data correspond to mean + SD. Asterisks (*) denote statistical significance (*P* < 0.05).

To assess whether cellular endogenous levels of BST-2 could restrict cell release, and thereby propagation, of LCMV progeny *in vitro*, we examined whether RNAi-mediated knock-down of BST-2 in HeLa cells, known to express high levels of BST-2 constitutively [31], affected cell release of LCMV particles. For this we transduced HeLa cells with a lentivirus expressing the pac gene that mediates resistance to puromycin and shRNAs to either BST-2 (HeLa-TKD), or a control shRNA (HeLa-pLKO). Transduced cells were cloned by limited dilution and selected in the presence of puromycin. We confirmed by FACS analysis that cell surface expression of BST-2 was greatly reduced in HeLa-TKD cells compared to control HeLa-pLKO cells (**Fig 1A**). We then infected HeLa-pLKO and HeLa-TKD cells with rLCMV/Z-FLAG [35], and at the indicated times post-infection (p.i.) we determined titers of cell-free virus infectious progeny (**Fig 1B**). At 48 hrs p.i. production of infectious LCMV was slightly higher (four-fold) in HeLa-TKD cells than HeLa-pLKO control cells. Consistent with this finding, we observed a slight increase in the numbers of viral antigen positive cells at 48 h p.i. in HeLa-TKD compared to HeLa-pLKO cells (**Fig 1C**). Expression levels of LCMV Z protein at 16 h p.i. were similar between HeLa-pLKO and HeLa-TKD cells (**Fig 1D**), suggesting that constitutive expression of BST-2 in HeLa cells did not significantly affect the translation efficiency of viral mRNAs or the early steps of the virus life cycle leading to the release of the vRNP into the cell cytoplasm where it directs virus RNA replication and gene transcription. We next examined whether the modest increase in production of infectious LCMV progeny in HeLa-TKD compared to HeLa-pLKO cells correlated with similar differences in production of total virion particles. For this, we infected HeLa-pLKO and HeLa-TKD cells with rLCMV/Z-FLAG, and at 24 hrs p.i. virion particles present in tissue culture supernatant were collected by ultracentrifugation and cell lysates prepared for protein analysis. Levels of Z protein in cell lysates and virions were determined by Western blot using an anti-FLAG antibody (Ab) (**Fig 1D and 1E**). Levels of Z protein in virion particle preparations were slightly higher (1.7-fold) in HeLa-TKD compared to control HeLa-pLKO cells, whereas lysates from infected HeLa-TKD and HeLa-pLKO cells had similar Z protein levels. Consistent with previous findings [39], we observed that VSV multiplication was enhanced by knock-down of BST-2 (**Fig 1G**).

**Fig 2:**
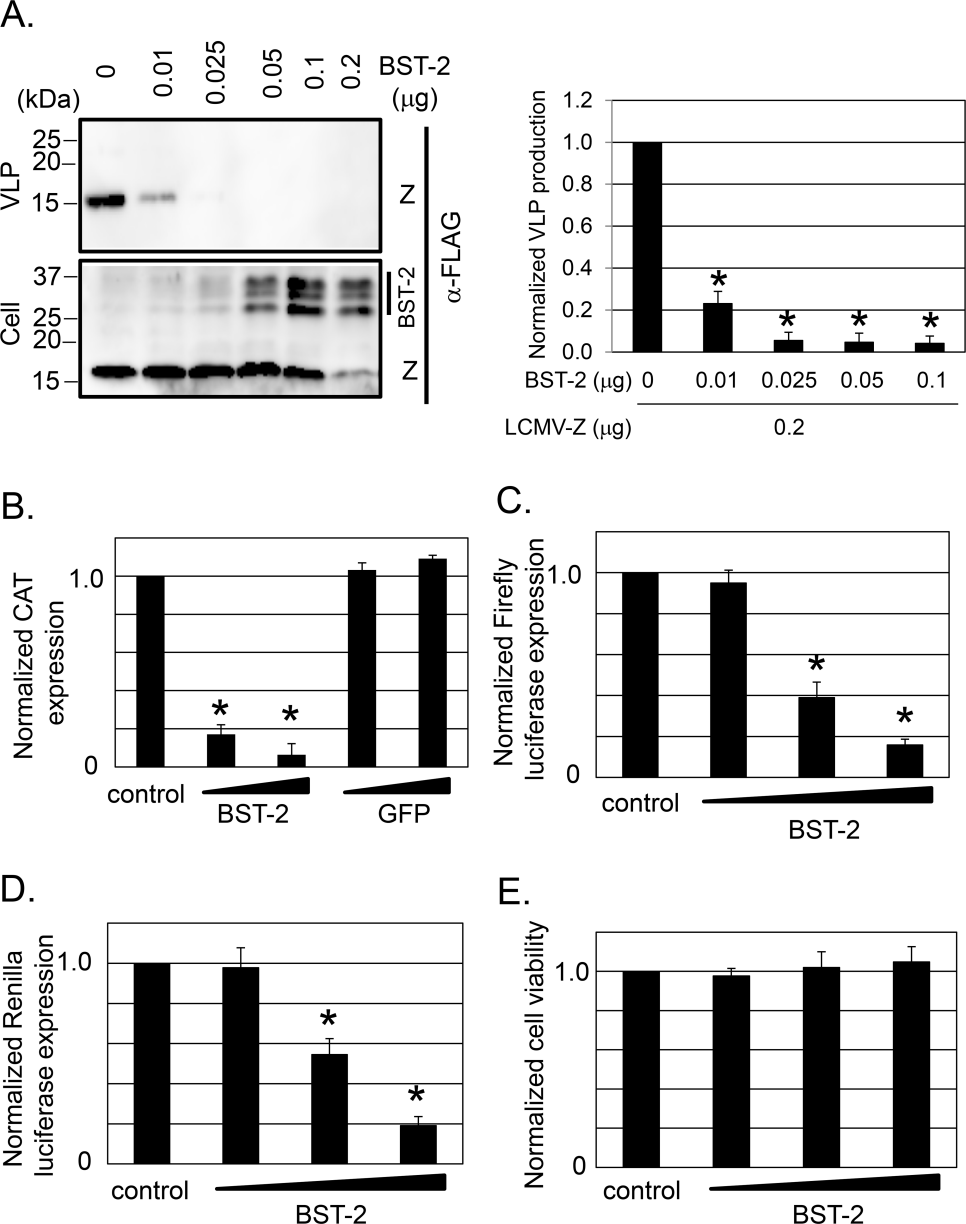
Effect of BST-2 on LCMV Z-mediated VLP production and vRNP activity. **A.** Effect of BST-2 expression on Z-mediated VLP production. 293T cells were transfected with pC-LCMV-Z-FLAG (0.2 μg) together with increasing amounts of pTeth-FL (BST-2). At 48 hrs post transfection, VLPs were recovered from TCS by ultracentrifugation and cell lysates prepared. Levels of Z protein present in VLPs and cell lysates, and BST-2 levels in cell lysates, were detected by WB using an Ab to FLAG. Normalized levels of VLP production were determined by assigning the value of 1 to the ratio of VLP/cell Z protein expression levels in cells transfected with pC-LCMV-Z-FLAG in the absence of BST-2 (n = 3; 2 independent experiments). **B.** Effect of BST-2 expression on LCMV vRNP activity. 293T cells were transfected with plasmids expressing the components of the LCMV MG-CAT, together with control BST-2 or GFP expression plasmids using the indicated plasmid DNA amounts. CAT expression was measured at 48 hrs post transfection. CAT expression levels were normalized by setting to 1 levels expressed by cells transfected with control plasmid. **C, D.** Effect of BST-2 over-expression on pol-II mediated expression. 293T cells were transfected with psiCHECK2 together with control plasmid (pcDNFL) or increasing amounts of BST-2 expression plasmid. At 48 hrs post transfection, cell lysates were prepared for detection of both Firefly luciferase (FL) (**C**) and Renilla luciferase (RL) (**D**) using a dual luciferase detection system. Expression levels of FL and RL were normalized by setting to 1 the expression levels obtained in cells transfected with FL or RL and the control plasmid. **E.** Effect of BST-2 expression on 293T cell viability. Cells were transfected with increasing amount of BST-2 expression plasmid, and at 48 hrs post transfection, cell viability was determined by Cell-Titer Glo (Material and Methods). Values were normalized by setting to 1 results from cells transfected with control plasmid (pcDNFL). Data correspond to mean + SD (three independent experiments). Asterisks denote statistical significance (*P < 0.05).

To investigate whether BST-2 interfered with cell release of LCMV particles, we examined the effect of BST-2 on the efficiency of Z-mediated virus-like particle (VLP) production using a well-established assay [2, 3]. For this we transfected 293T cells with a plasmid (0.2 μg) expressing a FLAG-tagged Z and increasing amounts of a plasmid expressing a FLAG-tagged BST-2, and at 48 h post transfection, we collected VLPs from tissue culture supernatants by ultracentrifugation and prepared cell lysates. Levels of Z protein present in VLPs and cell lysates, and BST-2 levels in cell lysates, were detected by Western blot using an antibody to FLAG. BST-2 exerted a dose-dependent inhibitory effect on VLP production (**Fig 2A**). It should be noted that we observed this inhibitory effect of BST-2 on Z-mediated VLP production only when we used low (≤ 0.1 μg) amounts of BST-2 expressing plasmid to transfect cells. The use of higher amounts of BST-2 expression plasmid resulted in a very dramatic reduction in intracellular expression levels of Z that prevented an accurate assessment of Z-mediated VLP production. We also examined whether BST-2 could directly affect the activity of the LCMV polymerase complex. For this we used an LCMV minigenome (MG) rescue assay [5]. We transfected 293T cells with optimized amounts of plasmids required for the intracellular reconstitution of an active LCMV vRNP that directs expression of the chloramphenicol acetyl transferase (CAT) reporter gene, together with increasing amounts of a BST-2 expressing plasmid. BST-2 exhibited a dose-dependent inhibitory effect on the expression levels of MG-directed chloramphenicol acetyltransferase (CAT) reporter gene expression (**Fig 2B**). The interpretation of this finding was complicated by our observation that BST-2 appears to generally inhibit pol-II mediated expression in transient transfection assays. This was evidenced by the reduced expression of the Firefly and Renilla luciferase genes, using psiCHECK2 plasmid, which possesses the Firefly luciferase gene driven by HSV-TK promoter and Renilla luciferase gene driven by SV40 promotor, respectively (**Fig 2C and 2D**). Increasing amount of exogenous BST-2 did not, however, affect cell viability **(Fig 2E)**.

To further investigate the effect of BST-2 over-expression on LCMV multiplication, we compared the propagation and production of LCMV infectious progeny between Huh7.5.1, which have non-detectable levels of BST-2, and Huh7.5.1 cells that constitutively express high levels of BST-2 (Huh7.5.1/BST2). To facilitate the assessment of cell-to-cell propagation of LCMV, we used a tri-segmented recombinant LCMV that expressed GFP (r3LCMV/GFP) [34]. Numbers of GFP positive cells were similar in both Huh7.5.1 and Huh7.5.1/BST2 cells at 24 hrs p.i. following challenge with different multiplicities of r3LCMV/GFP infection (moi = 0.001, 0.01, and 0.1) **(S1A Fig)**. In addition, both Huh7.5.1 and Huh7.5.1/BST2 cells exhibited similar kinetics **(S1B Fig)** and levels of viral RNA synthesis (both replication and transcription) following infection with LCMV as determined by Northern blot **(S1C Fig)**. We also examined the effect of BST-2 over-expression on production of LCMV infectious progeny in 293T cells. For this we transfected 293T cells with either BST-2 or control plasmid, followed by infection with LCMV (moi = 0.01) (**S2A Fig**). At 48 hrs p.i. we collected tissue culture supernatant to determine LCMV infectious titers. We observed a very modest (two-fold), statistically significant, decrease in production of infectious LCMV progeny by 293T cells that were transfected with BST-2 (**S2A Fig**). This experiment could have underestimated the inhibitory effect of BST-2 on LCMV release due to the contribution of viral titer from cells that did not get transfected. However, consistent with previous findings [39], we observed about five-fold reduction in VSV infectious titers produced by BST-2-transfected compared to control 293T cells transfected with pGFP (**S2B Fig**). Intriguingly, we observed that 293T cells over-expressing BST-2, but not GFP, became highly resistant to *de novo* LCMV infection (**S2C Fig**), which likely accounted for the modest reduction in production of infectious virus particles.

### Effect of LCMV infection on BST-2 expression levels and sub-cellular distribution in cultured cells

**Fig 3:**
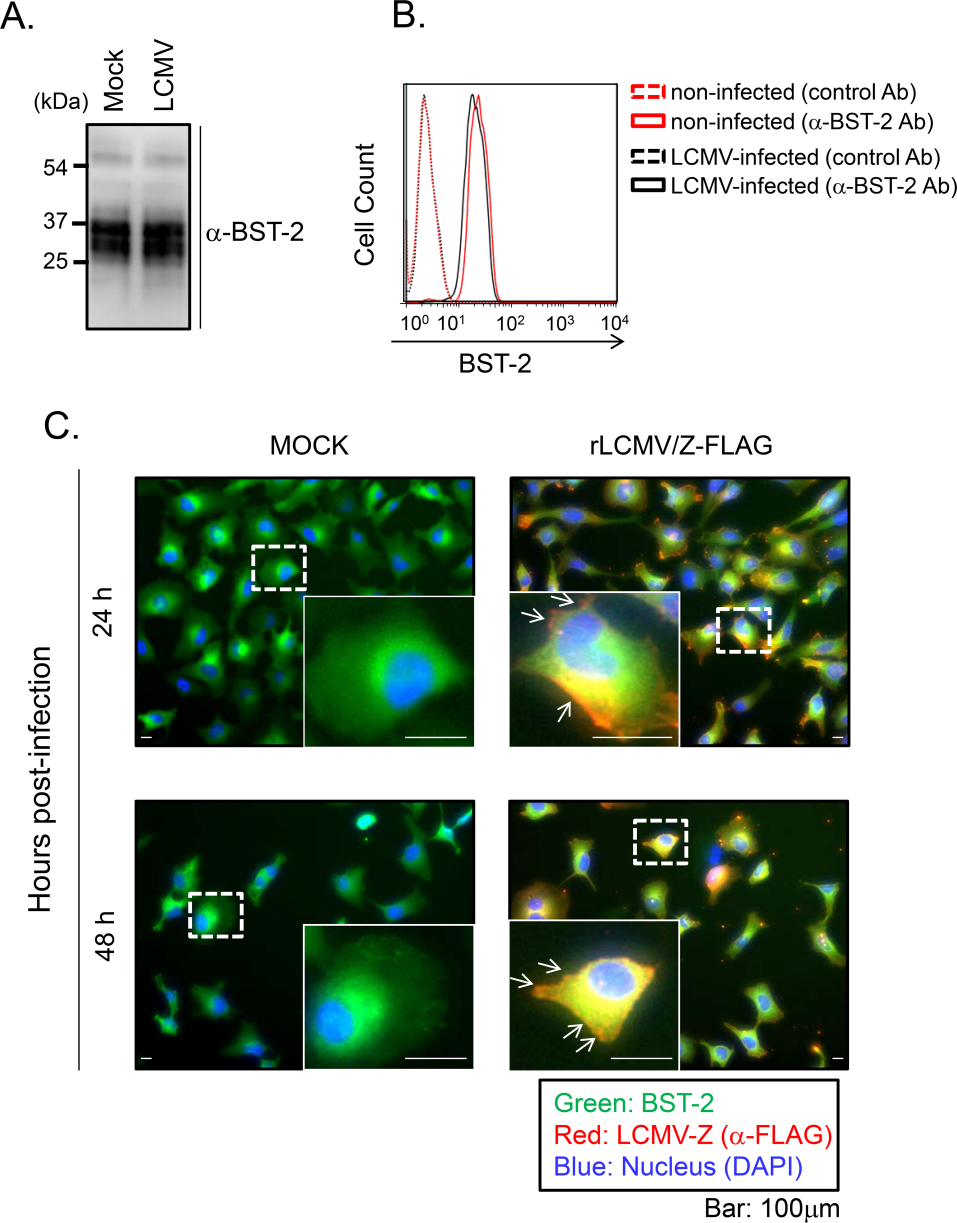
Effect of LCMV infection on protein expression and subcellular distribution of endogenous BST-2. **A.** HeLa cells were infected with LCMV (moi = 0.01). At 48 hrs p.i, cells were lysed and BST-2 expression was analyzed by WB using a polyclonal serum to α-BST-2. **B.** HeLa cells were infected with LCMV (moi = 0.01). At 48 hrs p.i, cells were reacted with either control antibody (Ab) or α-BST-2 Ab conjugated with PE and then fixed. Cell surface expression of BST-2 was analyzed by FACS Calibur (BD, San Jose, CA). **C.** Subcellular localization of BST-2 in HeLa cells infected with rLCMV/Z-FLAG. HeLa cells were infected with rLCMV/Z-FLAG (moi = 0.01). At 24 and 48 hrs p.i., cells were fixed with 4% PFA and stained with α-FLAG antibody and α-BST-2 antibody, followed with second antibodies conjugated with Alexa 568 or Alexa 488, respectively. Nuclei were stained with DAPI. White arrows indicate LCMV Z localized at the plasma membrane without BST-2 co-localization.

Several enveloped viruses have been shown to encode gene products that can counteract the antiviral activity of BST-2 via different mechanisms [13–16, 40], including down-regulation of BST-2 on the cell surface, degradation of intracellular BST-2, and sequestration of BST-2 in intracellular compartments different from the site where virion release takes place. We therefore examined whether LCMV infection affected the overall and cell surface BST-2 expression levels, or subcellular localization of BST-2. For this we infected HeLa cells with LCMV (moi = 0.01), and at 48 hrs p.i. we determined expression levels of BST-2 by Western blot using an anti-BST-2 Ab [31]. We did not observe differences in BST-2 expression levels between LCMV- and mock-infected control HeLa cells (**Fig 3A**). Likewise, we did not observe significant differences in cell surface expression of BST-2 between LCMV- and mock-infected control HeLa cells as determined by FACS analysis (**Fig 3B**). To assess whether LCMV infection altered the subcellular distribution of BST-2, we infected HeLa cells with rLCMV/Z-FLAG (moi = 0.01), and at 24 and 48 hrs p.i. we fixed the cells with 4% PFA and stained them with antibodies to both BST-2 and FLAG. Both rLCMV/Z-FLAG and mock-infected control cells exhibited the same subcellular distribution of BST-2, which localized predominantly to the peri-nuclear region in juxtaposition to the LCMV Z protein (**Fig 3C**).

### LCMV does not encode a BST-2 antagonist

To further confirm that LCMV infection does not antagonize BST-2-mediated restriction of virion release, we examined the effect of LCMV infection on production of Ebola virus (EBOV) matrix protein (VP40)-mediated VLP production, a process known to be inhibited by BST-2 expression [13, 41, 42]. 293T cells were transfected with plasmids expressing EBOV VP40 alone or together with BST-2, followed by infection with rLCMV/Z-FLAG (moi = 5). At 16 hrs p.i. we collected VLP-containing tissue culture supernatant and cells. After clarification from cell debris, we collected VLPs by ultracentrifugation and then analyzed cell lysates and VLPs by Western blot for VP40 expression. Ectopic over-expression of BST-2 significantly reduced EBOV VP40-mediated VLP production, a finding consistent with published data [41]. LCMV infection did not affect levels of VP40-mediated VLP production and did not alleviate BST-2 induced inhibition of VP40-mediated VLP production **(S3 Fig)**.

### BST-2 localizes to the splenic marginal zone / red pulp and confines a persistence prone strain of LCMV

**Fig 4:**
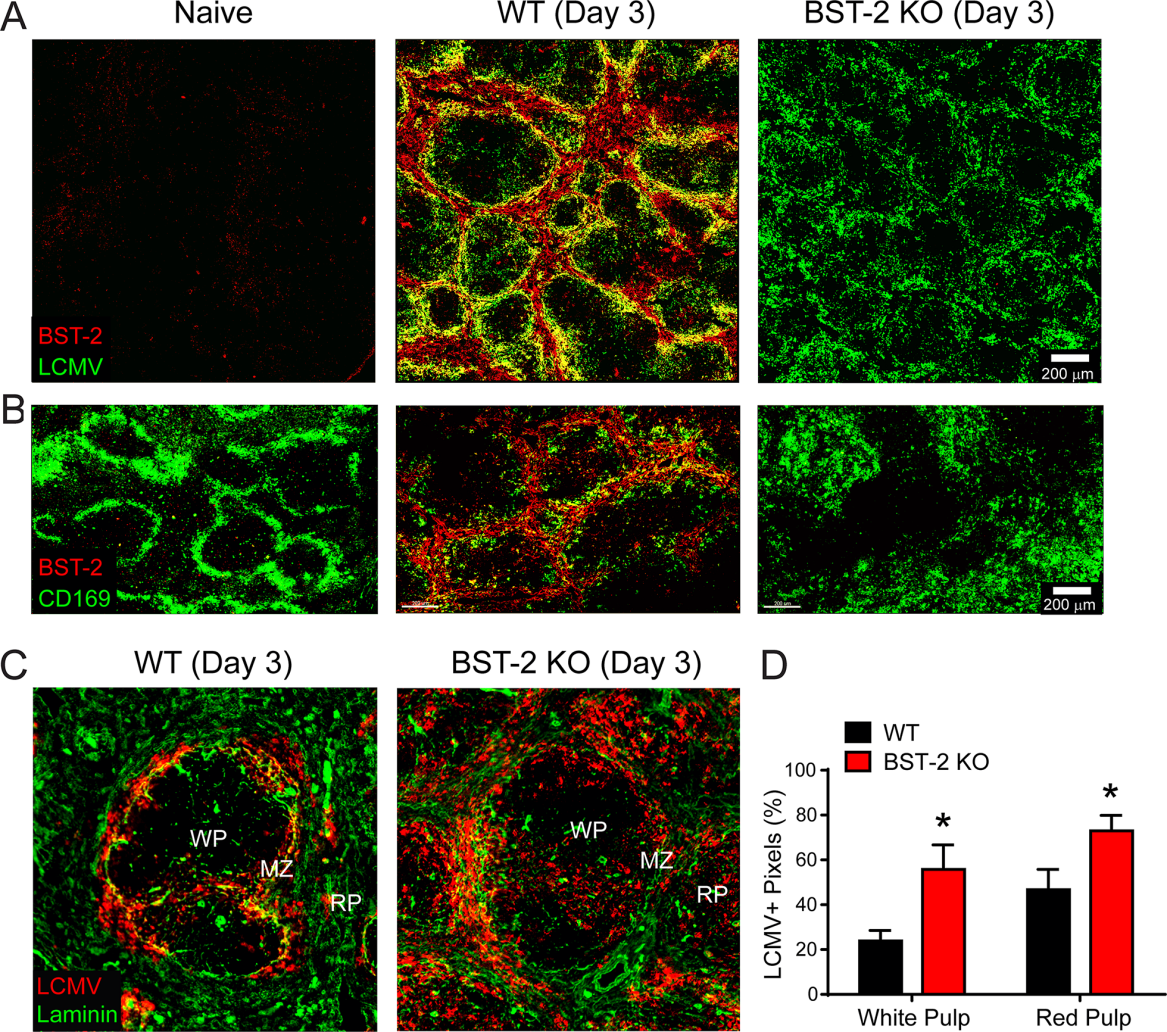
BST-2 contains LCMV within the splenic marginal zone. **A-B.** Representative confocal images from naïve, d3 WT, and d3 BST-2 KO mice depict the splenic distribution of LCMV Cl-13 (green; A) or CD169+ metallophillic macrophages (green; B) in relation to BST-2 expression (red) (n = 4 mice per group; 2 independent experiments). **C.** Representative confocal images captured in the spleens of WT vs. BST-2 KO mice at day 3 post-infection show the distribution of LCMV (red) in relation to the white pulp (WP), marginal zone (MZ), and red pulp (RP). Laminin staining is shown in green to delineate these anatomical regions. **D.** Quantification of the LCMV staining pattern shown in panel C (n = 8 mice per group; 2 independent experiments). Data are represented as mean ± SD. Asterisks denote statistical significance (*P < 0.05).

We next examined whether the modest effect of BST-2 on LCMV multiplication in cultured cells had any implications in the establishment of a persistent viral infection *in vivo*. To this end, we infected wild type (WT) and BST-2 knockout (KO) mice intravenously (i.v.) with LCMV clone 13 (Cl-13), a strain known to establish a chronic viral infection in immune competent adult mice [32, 33]. Immunohistochemical analysis of WT spleens at day 3 p.i. revealed increased expression of BST-2 in the marginal zone and red pulp relative to uninfected control mice and infected BST-2 KO mice (**Fig 4A**). Following i.v. inoculation, LCMV localizes to the splenic marginal zone [43], the area where we observed elevated BST-2 expression levels (**Fig 4A**). Further delineation of the anatomy was achieved using CD169 staining. CD169 is a cell adhesion molecule found on the surface of splenic marginal zone macrophages. CD169+ macrophages demarcated the ring of splenic BST-2 staining in WT mice at day 3 p.i. (**Fig 4B**). In addition to marginal zone macrophages, BST-2 was also expressed in B cells, CD4+ T cells, CD11b+ myeloid cells, and plasmacytoid DCs (pDCS) at this time point (**S4A Fig**).

Based on the anatomical expression pattern of BST-2 at early times after Cl-13 infection, we postulated that BST-2 might contribute to the early confinement of LCMV within the splenic marginal zone. To examine this hypothesis, we compared the anatomical distribution of Cl-13 in the spleens of WT vs. BST-2 KO mice at day 3 p.i. (**Fig 4C**). Consistent with our hypothesis, Cl-13 was no longer confined to the splenic marginal zone in BST-2 KO mice. Elevated expression of viral NP was observed in the splenic white and red pulp (**Fig 4C and 4D**), indicating that the virus had escaped from the marginal zone in the absence of BST-2.

### BST-2 deficiency promotes a functional defect in LCMV-specific T cells

**Fig 5:**
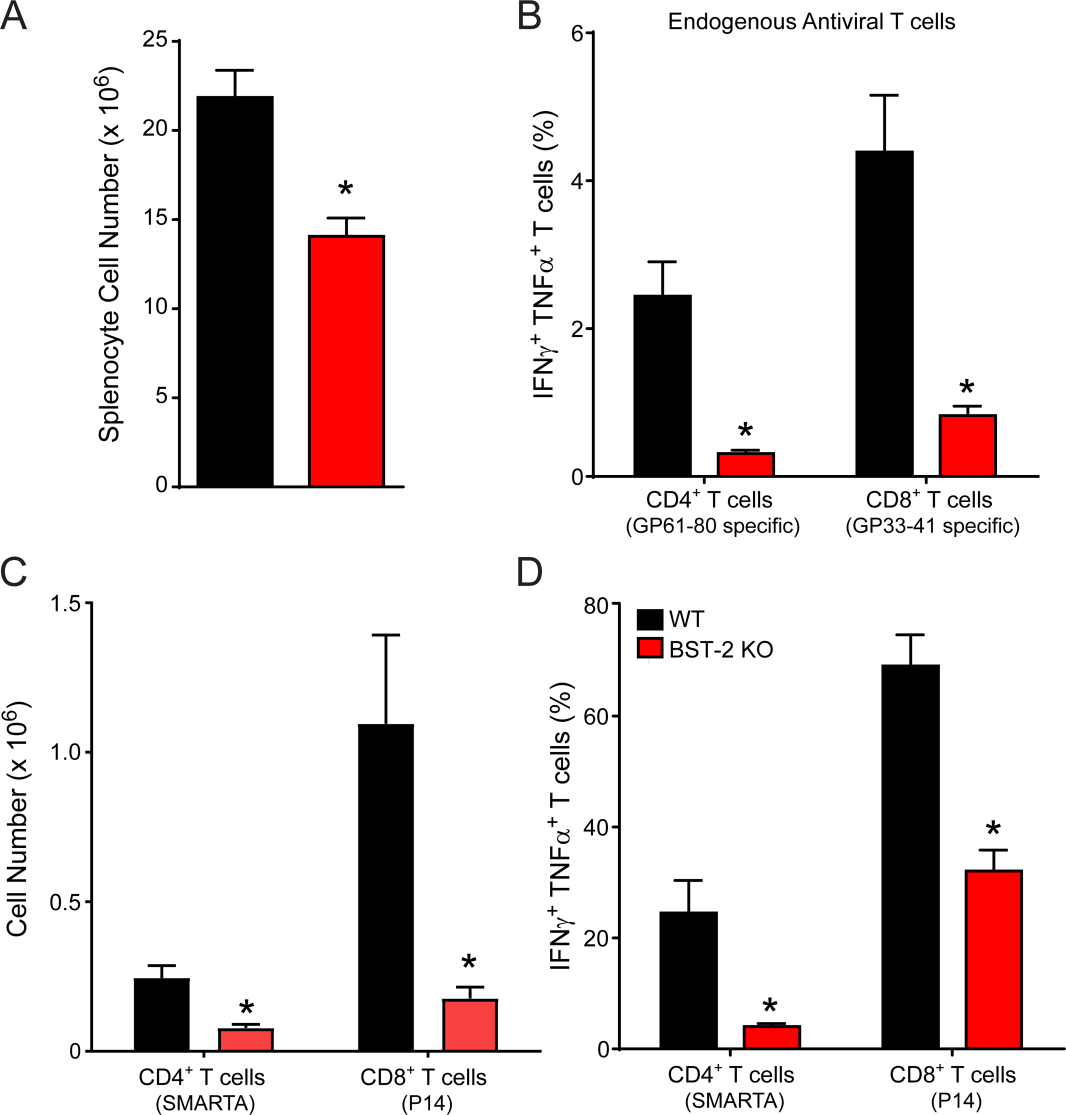
Antiviral T cell function and expansion is reduced in BST-2 deficient mice. **A-D.** Naïve WT and BST-2 KO mice that received 2,000 Thy1.1+ P14 and 2,000 CD45.1+ SMARTA cells were infected with LCMV Cl-13. On day 8 post-infection, the absolute number of splenocytes (A), P14 cells (C), and SMARTA (C) cells was quantified. In addition, the percentage of endogenous (B) and transferred (D) IFNγ+ TNFα+ antiviral T cells was determined following a 5 hr *in vitro* stimulation with GP33-41 (CD8) or GP61-80 (CD4) peptide (n = 9 mice per group; 2 independent experiments). Data are represented as mean ± SD. Asterisks denote statistical significance (*P < 0.05).

Because early viral control is a critical component in the development of successful adaptive immune responses, we next examined the impact of BST-2 deficiency on the development of LCMV-specific CD8+ and CD4+ T cell responses. As surrogates for the anti-viral T cell response, we selected two well-described immunodominant peptides for *ex vivo* stimulation assays: GP33-41 (CD8) and GP61-80 (CD4) [37, 38]. Prior to infection, most splenic immune cell populations were comparable between WT and BST-2 KO mice (**S4B Fig**). In BST-2 KO mice at day 8 p.i., we observed a significant reduction in the absolute number of splenocytes (**Fig 5A**), already suggesting a reduction in T cell clonal expansion. This was confirmed by our *ex vivo* peptide stimulation assays, which revealed a significant reduction in the percentage of endogenous IFNγ+ TNFα+ GP61-80-specific CD4+ T cells and GP33-41-specific CD8+ T cells in BST-2 KO mice relative to WT controls (**Fig 5B**). To ensure that this defect was not linked to a T cell intrinsic function of BST-2, we adoptively transferred a combination of 2,000 WT D^b^GP33-41 Thy1.1+ CD8+ T cells and 2,000 I-A^b^GP61-80 CD45.1+ CD4+ T cells i.v. into WT and BST-2 KO mice. These T cell receptor transgenic cell populations are commonly referred to as P14 [18] and SMARTA cells [20], respectively. One day following injection of naïve P14 and SMARTA cells, mice were infected with Cl-13. As with the endogenous anti-viral T cell response, the absolute number (**Fig 5C**) and function (**Fig 5D**) of both P14 and SMARTA cells were significantly reduced in day 8 BST-2 KO mice relative to WT controls. These data indicate that the effect of BST-2 deficiency on the adaptive response is T cell extrinsic.

### Effect of BST-2 on location and proliferation of virus-specific T cells

**Fig 6:**
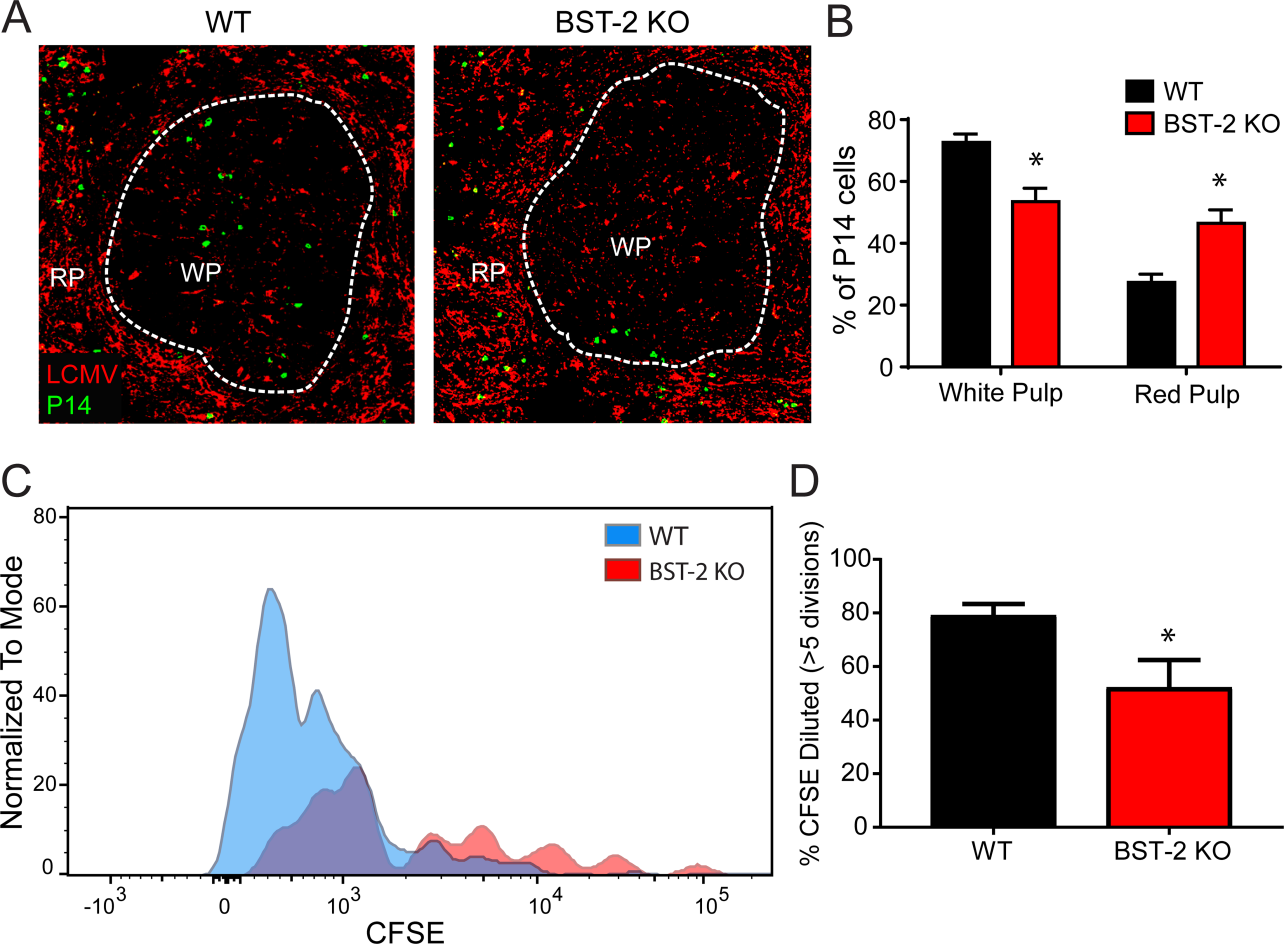
BST-2 deficiency changes the splenic distribution and proliferative capacity of antiviral CD8+ T cells. **A.** Representative confocal images were captured at day 4 post-infection in the spleens of WT and BST-2 KO mice seeded with mOrange+ P14 cells (green). The splenic distribution of P14 cells in relation to LCMV Cl-13 (red) is shown. The white dotted line demarcates the border between the white pulp (WP) and red pulp (RP). **B.** The bar graph shows quantification of the white vs. red pulp P14 percentage in WT vs. BST-2 KO mice (n = 4 mice per group; 2 independent experiments). Data are represented as mean ± SD. Asterisks denote statistical significance (*P < 0.05). **C.** The representative histogram depicts the dilution of CFSE in P14 cells from WT (blue) vs. BST-2 KO (red) mice at day 3 post-infection. **D.** The bar graph shows quantification of the CFSE dilution data in panel C (n = 4 mice per group; 2 independent experiments). Graphed are the percentage of P14 cells that divided more than 5 times. Data are represented as mean ± SD. Asterisks denote statistical significance (*P ≤ 0.05).

The impact of BST-2 deficiency on LCMV-specific T cell function at day 8 post-infection was quite significant. We therefore assessed whether this dysfunction was set into motion at earlier time points post-infection. The splenic red pulp and marginal zone are known to express an abundance of immunoregulatory molecules (e.g. IL-10 and PD-L1) following Cl-13 infection [44, 45], and we postulated that early viral movement into these regions could draw anti-viral T cells away from the splenic white pulp where they are normally primed. To address this possibility, we adoptively transferred naïve mOrange+ P14 cells into WT and BST-2 KO, and quantified their anatomical distribution in the spleen at day 4 p.i. Interestingly, we observed a higher proportion of P14 CD8+ T cells in the splenic red pulp, and a corresponding decrease in the white pulp, of BST-2 KO mice relative to WT controls (**Fig 6A and 6B**). We also observed a decreased percentage of carboxyfluorescein succinimidyl ester (CFSE) diluted P14 cells in spleens of BST-2 KO mice at this same time point, indicative of reduced proliferation (**Fig 6C and 6D**). These data demonstrate that virus-specific CD8+ T cells mis-localize in the spleens of BST-2 KO mice, which likely causes them to proliferate less during the critical early stages of T cell priming.

### BST-2 promotes control of acute and persistent LCMV infections

**Fig 7:**
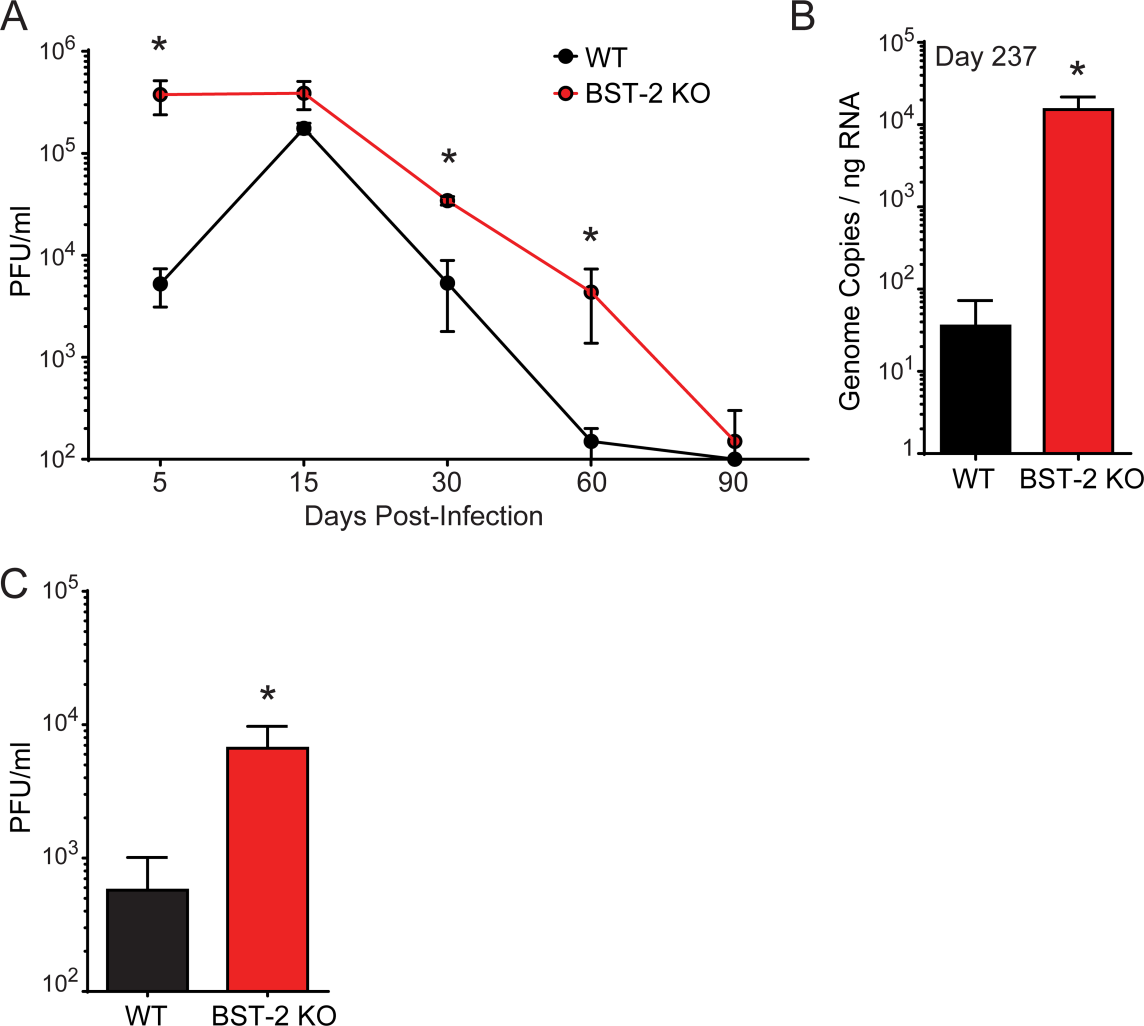
BST-2 deficiency impedes control of acute and persistent strains of LCMV. **A.** Serum viral titers were quantified by plaque assay in WT vs. BST-2 KO mice infected with LCMV Cl-13 (n = 5 mice per group; 2 independent experiments). **B.** Brain viral loads were quantified by Q-PCR [48] in WT vs. BST-2 KO mice 237 days following infection with LCMV Cl-13 (n = 3–4 mice per group; 2 independent experiments). **C.** Serum viral titers were quantified by plaque assay in WT vs. BST-2 KO mice 5 days following infection with LCMV Arm (n = 3–4 mice per group; 2 independent experiments). All data in this figure are represented as mean ± SD. Asterisks denote statistical significance (*P < 0.05).

The dysfunctional anti-viral T cell response observed in BST-2 KO mice led us to ultimately assess the impact this has on viral control *in vivo*. Cl-13 is known to persist in most peripheral tissues, including the blood, until ~day 60 p.i. [46]. In BST-2 KO mice, we observed elevated LCMV clone 13 titers in the blood beginning at day 5, and at all time points measured thereafter, until the virus was ultimately controlled at day 90 (**Fig 7A**). Control of Cl-13 in BST-2 KO mice required 30 additional days compared to WT mice (**Fig 7A**). The brain can serve as a reservoir for long term viral persistence in Cl-13-infected WT mice [47]. Interestingly, viral titers remained significantly elevated in the brains of BST-2 KO at nearly 8 months p.i. (**Fig 7B**). Lastly, we tested whether BST-2 also plays a role in controlling an acute LCMV infection. LCMV Armstrong (Arm) differs from Cl-13 by 3 amino acids, and ARM is usually cleared within a week of i.v. inoculation, and is difficult to detect in the blood at any time point p.i. [32, 33, 46]. However, in BST-2 KO mice, we noted significantly elevated titers of LCMV Arm in the blood at day 5 p.i. (**Fig 7C**). These results indicate that BST-2 plays a role in controlling LCMV *in vivo*.

## Discussion

Innate immunity is the first line of host defense against viral infections. Accordingly, to facilitate the completion of a successful infection many viruses encode molecules that counteract different components of the host innate immune response. Arenavirus NP has been shown to interfere with both induction of IFN-I [49–52] and activation of NF-kB, a critical player in the host inflammatory response to infection [53, 54]. Likewise, we have documented that the IFN-I inducible BST-2 interferes with LASV Z/GPC-induced VLP production [22]. Similar to that described for HIV-1 [22, 25], BST-2 appears to retain LASV VLP on the cell surface. Consistent with this observation, BST-2 was reported to inhibit production of LASV infectious progeny [55]. However, whether these findings are unique to LASV or generally applicable to arenaviruses, remains unknown. In this study, we investigated the contribution of BST-2 to the antiviral defense mounted against the prototypic arenavirus, LCMV.

Knock-down of BST-2 in HeLa cells (**Fig 1A**), known to express a constitutively high level of BST-2, caused a reduction, although very modest, on production of infectious progeny (**Fig 1B**) that correlated only partially with a similar minimal reduction in production of virus particles **(Fig 1C)**. BST-2 did not affect LCMV entry, virus RNA replication, transcription, or viral mRNA translation efficiency (**Fig 1D**). These findings suggested, as documented for other viruses [11, 22, 55], that BST-2 might also affect LCMV cell release, a process driven by the LCMV Z protein [2]. To evaluate this hypothesis, we examined the effect of BST-2 on Z-mediated VLP release and found that BST-2 exhibited a dose-dependent inhibitory effect on Z-mediated VLP release (**Fig 2A**), a finding consistent with those documented for LASV [22] and other enveloped viruses [11, 12, 41, 55]. Unexpectedly, BST-2 inhibited in a dose-dependent manner replication and expression of an LCMV mini genome (MG) (**Fig 2B**). However, the interpretation and significance of this finding is confounded by our finding that in cell transfection assays, BST-2 exhibited a dose-dependent inhibitory effect on expression of co-transfected Renilla or Firefly luciferase, suggesting that under our experimental conditions, BST-2 expression might interfere with RNA Pol-II polymerase mediated gene expression (**Fig 2C and 2D**), which is consistent with other published results [56]. Likewise, 293T cells over-expressing plasmid supplied BST-2 exhibited a rather modest restriction to LCMV multiplication (**S2A Fig**). Unexpectedly, we observed that 293T cells expressing very high levels of BST-2 were rather refractory to LCMV infection (**S2C Fig**), a finding that might have accounted for the minor differences in production of LCMV infectious progeny between BST-2 expressing and control 293T cells.

Constitutive expression of BST-2 in Huh7.5.1 did not have any significant effect on LCMV propagation and production of infectious LCMV progeny (**S1A Fig and S1B Fig**). Accordingly, LCMV RNA synthesis, both genome replication and gene transcription, were not affected in Huh7.5.1/BST2 compared to Huh7.5.1 cells (**S1C Fig**). The apparent discrepancy between observations presented in **S1 Fig** and **S2 Fig** is best explained by the significantly higher expression levels of BST-2 in transfected 293T cells compared to Huh7.5.1/BST-2 cells.

Several enveloped viruses encode molecules that counteract the antiviral activity of BST-2 via a variety of mechanisms. For example, HIV-1 Vpu induces degradation of BST-2 [11, 12], whereas Ebola virus GP sequesters BST-2 from the virus budding membrane region [13, 42, 57–61]. Cell surface expression, overall protein expression, and subcellular localization of BST-2 were not affected upon LCMV infection (**Fig 3**). This in addition to the inability of LCMV infection to counteract the BST-2-mediated inhibitory effect on the budding activity of VP40 (**S3 Fig**) further supported our conclusion that LCMV does not encode a BST-2 antagonist.

Despite the relatively modest effect of BST-2 on LCMV *in vitro*, the absence of this antiviral protein played a critical role in shaping the early viral distribution and subsequent immune defense *in vivo*. Following intravenous inoculation, LCMV localizes initially to the splenic marginal zone [43]. This area is inhabited by CD169+ marginal zone macrophages that can capture materials from the blood and bear resemblance to subcapsular sinus macrophages in draining lymph nodes [62]. The speed and efficiency with which LCMV moves from marginal zone macrophages into the surrounding white and red pulp likely influences its ability to establish persistence. Interestingly, we observed elevated BST-2 expression in the marginal zone region where LCMV Cl-13 localized early after infection (**Fig 4**). This is consistent with local induction of BST-2 synthesis by IFN-I [63]. The functional importance of early expression of this antiviral protein during LCMV infection was established in BST-2 KO mice, where we observed a failure to strictly confine LCMV Cl-13 to the splenic marginal zone at day three post-infection. BST-2 is known to have properties that promote immune function [17, 64–66], but this failure to confine LCMV Cl-13 is most consistent with the ability of BST-2 to sequester or “tether” viruses [63].

BST-2 is known to affect replication of some viruses [17, 63, 67–71], but not others [72, 73] *in vivo*. This is likely due to the ability of BST-2 to bind specific viral proteins, the degree of IFN-I induced BST-2 synthesis, and the role of BST-2 in directly promoting antiviral immune responses. The contribution of BST-2 to the immune defense against LCMV Cl-13 is intriguing, because its absence induced profound antiviral T cell dysfunction in a cell extrinsic manner (**Fig 5**). LCMV Cl-13 is known to induce immune exhaustion in LCMV-specific CD8+ and CD4+ T cells [32, 74, 75], but this functional exhaustion was significantly elevated in BST-2 KO mice. In addition, BST-2 deficiency reduced the ability of wild type LCMV-specific CD8+ T cells to proliferate, which was likely due to elevated splenic viral titers and the early migration of CD8+ T cells into the splenic red pulp (**Fig 6**) and / or faulty priming by BST-2 KO dendritic cells [65, 66]. The splenic red pulp is known to express high levels of immunoregulators (e.g. IL-10 and PD-L1) that suppress antiviral T cell cytokine production and proliferation [44, 45]. We postulate that BST-2 plays an important role in filter organs like the spleen by confining circulating viruses to the splenic marginal zone. This gives the adaptive immune system time to respond and eventually eradicate the pathogen. However, in the absence of BST-2, a persistence prone virus like LCMV Cl-13 gained an advantage by escaping from the marginal zone more quickly. In fact, serum viral titers were significantly elevated in BST-2 KO mice, even though Cl-13 was eventually controlled in the serum by day 90 (**Fig 7**). Titers remained elevated in the brain, which is considered a site of long term persistence following LCMV Cl-13 infection [47]. We also observed that a non-persistent strain of LCMV (Armstrong) had an advantage in BST-2 KO mice.

Collectively, these data demonstrate BST-2 is an important component of the IFN-I inducible defense against LCMV. By interfering with the budding process mediated by the LCMV Z protein, BST-2 appears to slow down the spread of this virus both *in vitro* and *in vivo*. While the inhibition observed *in vitro* was modest, the antiviral impact of this protein was amplified into a much larger effect *in vivo*. These findings highlight the importance of sequestering viruses to specific anatomical compartments *in vivo*. Viral confinement to regions like the splenic marginal zone can shape the ensuing adaptive immune response and influence states of persistence. It will be important in future studies to determine whether the spread of other more pathogenic arenaviruses (e.g. LASV and JUNV) are similarly influenced by BST-2.

## Supporting information

S1 Fig Constitutive and transient expression of BST-2 does not affect LCMV multiplication. **A.** Propagation of LCMV. Huh7.5.1 and Huh7.5.1/BST2 cells were infected with r3LCMV/GFP at the indicated moi, and at 24 hrs p.i. cells were fixed and GFP positive cells visualized by epifluorescence. **B.** Production of infectious LCMV progeny. Huh7.5.1 and Huh7.5.1/BST2 cells were infected with LCMV at the indicated moi and at the indicated times p.i., titers of infectious LCMV in TCS were determined. **C.** LCMV RNA synthesis. Huh7.5.1 and Huh7.5.1/BST2 cells were infected with LCMV (moi = 0.1) and at the indicated times p.i., total cellular RNA was isolated and analyzed by Northern blot hybridization using an LCMV NP DNA probe that recognized S genome (replication) and NP mRNA (transcription) RNA species.
(TIF)

S2 Fig Effect of BST-2 over-expression on LCMV multiplication. **A-B.** 293T cells were transfected with either pcDNFL (Control), pTeth-FL (BST-2) or pGFP. At 12 hrs post transfection, cells were infected with either LCMV (moi = 0.01) or VSV (moi = 0.2) and 48 (LCMV infection) or 24 (VSV infection) hrs p.i. LCMV (**A**) and VSV (**B**) titers in TCS were determined by plaque assay (**A**, n = 3, 2 independent experiments; **B**, 3 independent experiments). Data correspond to mean + SD. Asterisks (*) denote statistical significance (*P* < 0.05). **C.** 293T cells were transfected with either pTeth-FL or pGFP and 12 hrs later infected with LCMV. At 36 hrs p.i. cells were fixed (4% PFA) stained with antibodies to LCMV NP and BST-2. Nuclei were visualized by DAPI staining.
(TIF)

S3 Fig LCMV infection does not rescue BST-2 induced inhibition of EBOV VP40-mediated VLP production. **A.** 293T cells were transfected with pCEboZVP40 and either control plasmid (pcDNFL) or pTeth-FL. At 5 hrs post-transfection, cells were infected with rLCMV/Z-FLAG (moi = 5). At 16 hrs post-infection cell- and VLP-associated VP40 protein expression levels were determined by WB. Levels of BST-2 and actin in cell lysates were also determined by WB. **B.** The ratio of VLP/cell of VP40 protein levels in cells transfected with control plasmid was set to 1.0 (n = 6; 2 independent experiments). Data correspond to mean + SD. Asterisks (*) denote statistical significance (*P* < 0.05).
(TIF)

S4 Fig Quantification BST-2-expressing cells and splenic immune cell subsets. **A.** The identity of BST-2-expressing splenic immune cells was determined flow cytometrically in WT mice 3 days following LCMV Cl-13 infection. FACs analysis was used to gate LIVE CD45+ BST-2+ cells in WT mice. Positive BST-2 signal was determined by comparing staining in WT vs. BST-2 KO mice. We then calculated the percentage of BST-2 expressing cells that were B cells (B220+ CD11c-), myeloid cells (B220- CD11b+), CD4+ T cells (B220- CD11b- CD4+), and pDCs (B220+ CD11c+). These subsets accounted for all but 4.3% of the BST-2-expressing cells (n = 5 mice per group). **B.** The absolute number of LIVE CD45+ B cells (CD19+), CD4+ T cells (Thy1.2+ CD4+), CD8+ T cells (Thy1.2+ CD8+), CD11b+ DCs (Thy1.2- CD19- CD11c+ CD11b+), CD8+ DCs (Thy1.2- CD19- CD11c+ CD8+), pDCs (Thy1.2- CD19- CD11c+ CD11b- B220+), and monocytes / macrophages (Thy1.2- CD19- CD11c- CD11b+) was determined flow cytometrically in the spleens of naïve WT vs. BST-2 KO mice (n = 5 mice per group). Data are represented as the mean + SD. Asterisks (*) denote statistical significance (*P* < 0.05).
(TIF)
