# Supplementary figures and images for: BST-2 controls T cell proliferation and exhaustion by shaping the early distribution of a persistent viral infection
Source: PLoS Pathog. 2018 Jul 20;14(7):e1007172. doi: 10.1371/journal.ppat.1007172 (PMC6080785; doi:10.1371/journal.ppat.1007172)

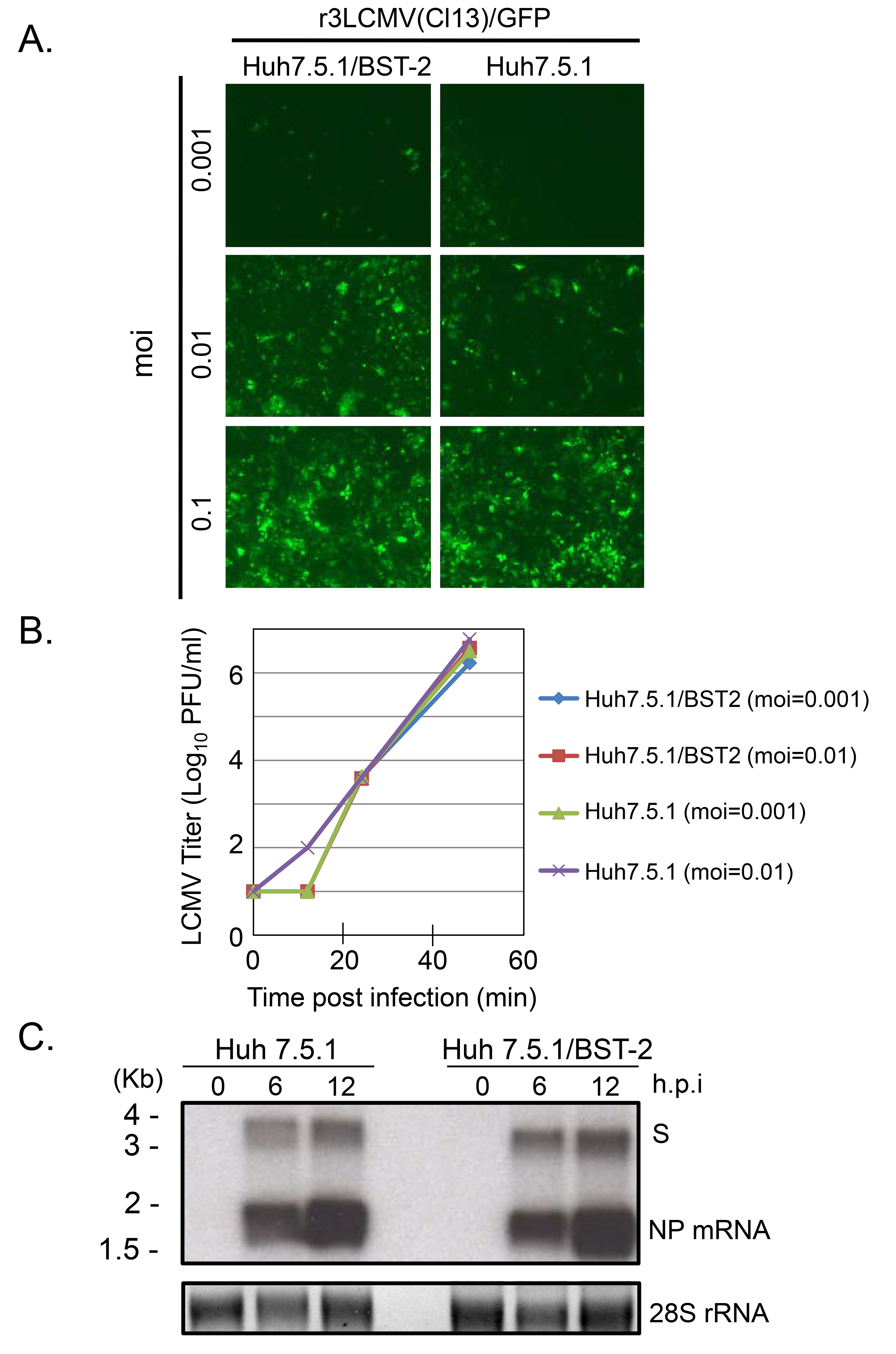

Supplement: S1 Fig — A. Propagation of LCMV. Huh7.5.1 and Huh7.5.1/BST2 cells were infected with r3LCMV/GFP at the indicated moi, and at 24 hrs p.i. cells were fixed and GFP positive cells visualized by epifluorescence. B. Production of infectious LCMV progeny. Huh7.5.1 and Huh7.5.1/BST2 cells were infected with LCMV at the indicated moi and at the indicated times p.i., titers of infectious LCMV in TCS were determined. C. LCMV RNA synthesis. Huh7.5.1 and Huh7.5.1/BST2 cells were infected with LCMV (moi = 0.1) and at the indicated times p.i., total cellular RNA was isolated and analyzed by Northern blot hybridization using an LCMV NP DNA probe that recognized S genome (replication) and NP mRNA (transcription) RNA species. (TIF) [file ppat.1007172.s001.tif]

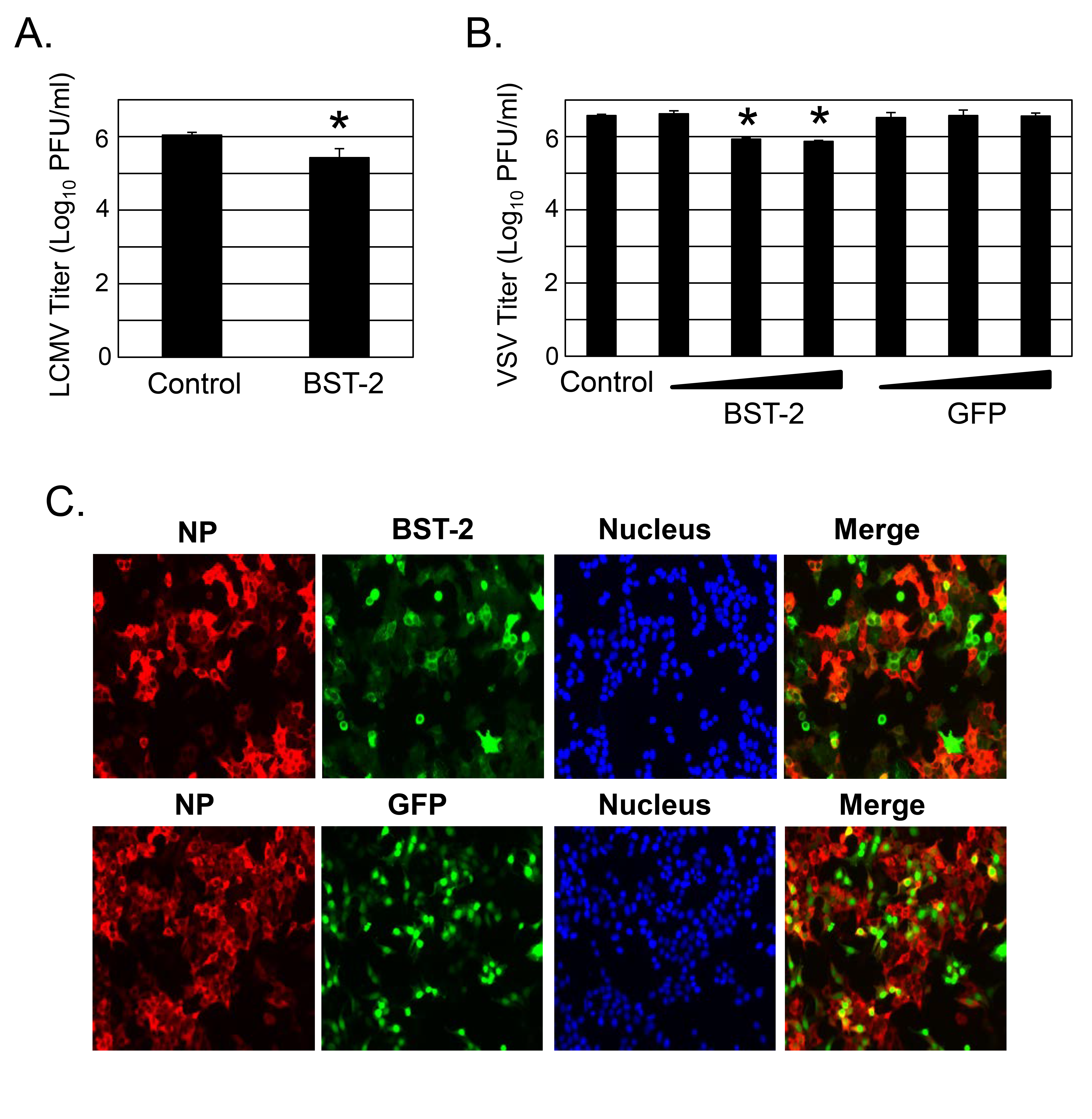

Supplement: S2 Fig — A-B. 293T cells were transfected with either pcDNFL (Control), pTeth-FL (BST-2) or pGFP. At 12 hrs post transfection, cells were infected with either LCMV (moi = 0.01) or VSV (moi = 0.2) and 48 (LCMV infection) or 24 (VSV infection) hrs p.i. LCMV (A) and VSV (B) titers in TCS were determined by plaque assay (A, n = 3, 2 independent experiments; B, 3 independent experiments). Data correspond to mean + SD. Asterisks (*) denote statistical significance (P < 0.05). C. 293T cells were transfected with either pTeth-FL or pGFP and 12 hrs later infected with LCMV. At 36 hrs p.i. cells were fixed (4% PFA) stained with antibodies to LCMV NP and BST-2. Nuclei were visualized by DAPI staining. (TIF) [file ppat.1007172.s002.tif]

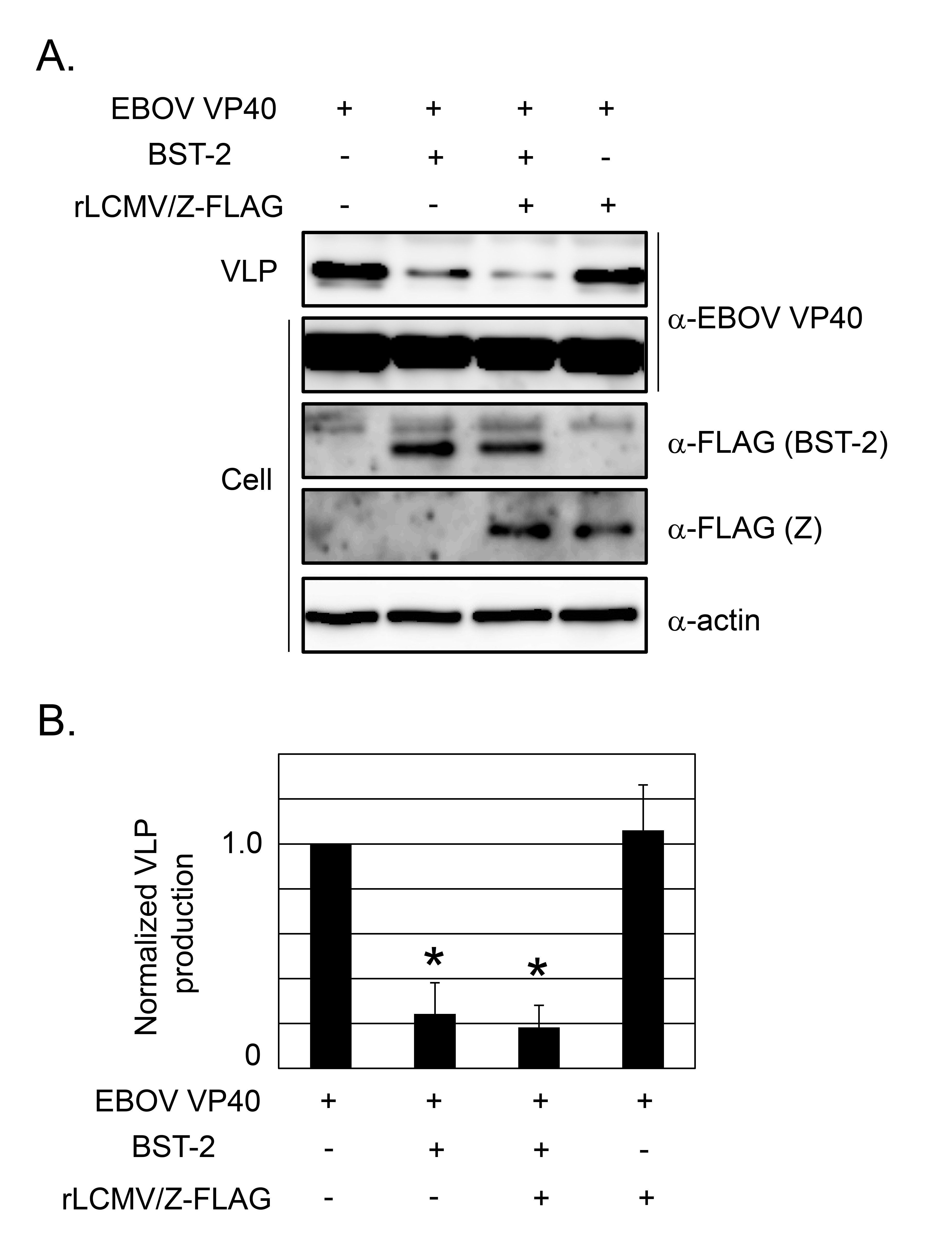

Supplement: S3 Fig — A. 293T cells were transfected with pCEboZVP40 and either control plasmid (pcDNFL) or pTeth-FL. At 5 hrs post-transfection, cells were infected with rLCMV/Z-FLAG (moi = 5). At 16 hrs post-infection cell- and VLP-associated VP40 protein expression levels were determined by WB. Levels of BST-2 and actin in cell lysates were also determined by WB. B. The ratio of VLP/cell of VP40 protein levels in cells transfected with control plasmid was set to 1.0 (n = 6; 2 independent experiments). Data correspond to mean + SD. Asterisks (*) denote statistical significance (P < 0.05). (TIF) [file ppat.1007172.s003.tif]

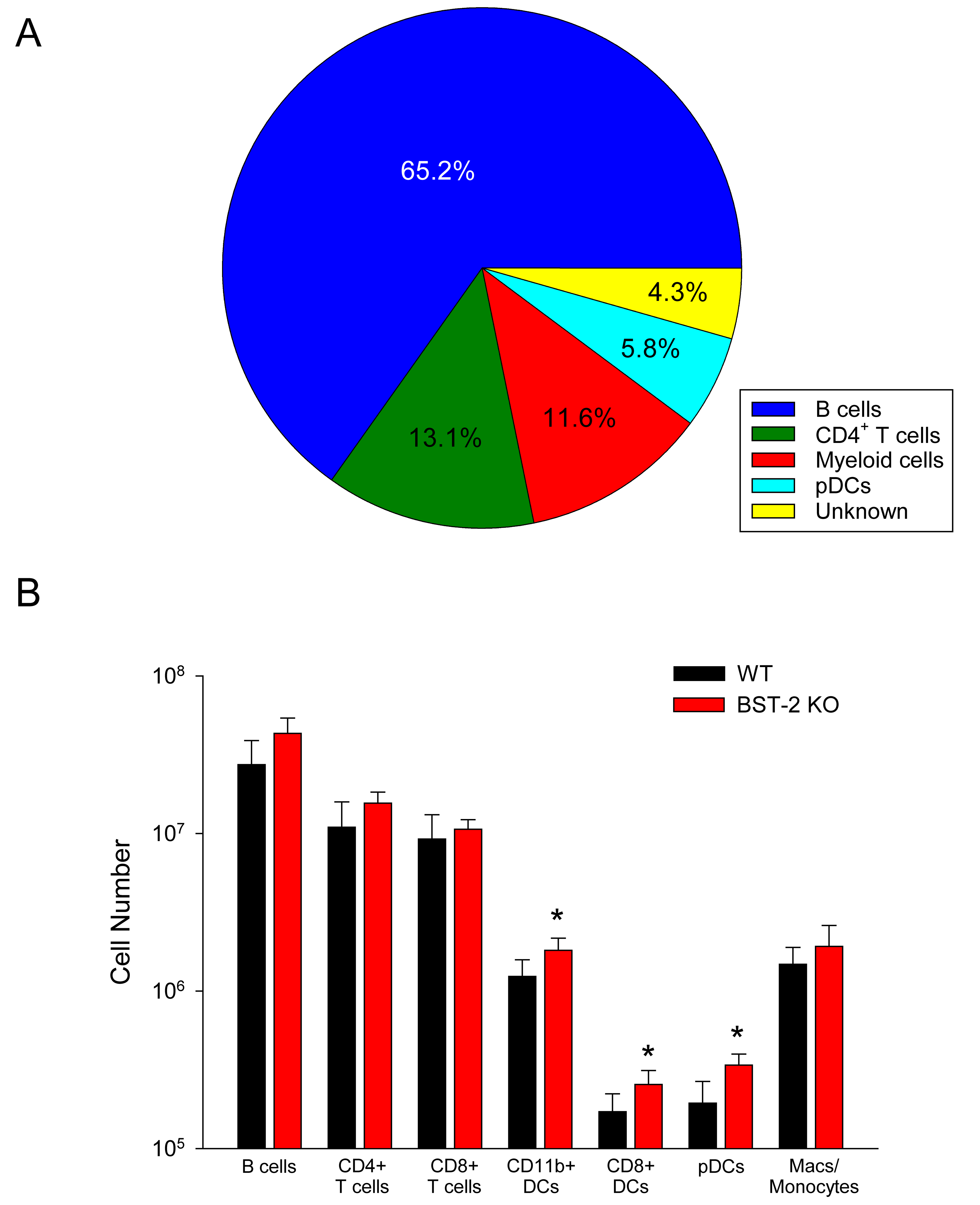

Supplement: S4 Fig — A. The identity of BST-2-expressing splenic immune cells was determined flow cytometrically in WT mice 3 days following LCMV Cl-13 infection. FACs analysis was used to gate LIVE CD45+ BST-2+ cells in WT mice. Positive BST-2 signal was determined by comparing staining in WT vs. BST-2 KO mice. We then calculated the percentage of BST-2 expressing cells that were B cells (B220+ CD11c-), myeloid cells (B220- CD11b+), CD4+ T cells (B220- CD11b- CD4+), and pDCs (B220+ CD11c+). These subsets accounted for all but 4.3% of the BST-2-expressing cells (n = 5 mice per group). B. The absolute number of LIVE CD45+ B cells (CD19+), CD4+ T cells (Thy1.2+ CD4+), CD8+ T cells (Thy1.2+ CD8+), CD11b+ DCs (Thy1.2- CD19- CD11c+ CD11b+), CD8+ DCs (Thy1.2- CD19- CD11c+ CD8+), pDCs (Thy1.2- CD19- CD11c+ CD11b- B220+), and monocytes / macrophages (Thy1.2- CD19- CD11c- CD11b+) was determined flow cytometrically in the spleens of naïve WT vs. BST-2 KO mice (n = 5 mice per group). Data are represented as the mean + SD. Asterisks (*) denote statistical significance (P < 0.05). (TIF) [file ppat.1007172.s004.tif]
